# Digestive Ability, Physiological Characteristics, and Rumen Bacterial Community of Holstein Finishing Steers in Response to Three Nutrient Density Diets as Fattening Phases Advanced

**DOI:** 10.3390/microorganisms8030335

**Published:** 2020-02-27

**Authors:** Qinghua Qiu, Chaoyu Gao, Muhammad Aziz ur Rahman, Binghai Cao, Huawei Su

**Affiliations:** 1 State Key Laboratory of Animal Nutrition, College of Animal Science and Technology, China Agricultural University, Beijing 100193, China; rcauqqh@cau.edu.cn (Q.Q.);; 2 Institute of Animal and Dairy Sciences, University of Agriculture, Faisalabad 35200, Pakistan

**Keywords:** ambient temperature, dietary nutrient density, nutrient digestibility, plasma metabolism, rumen bacterial community, rumen fermentation

## Abstract

The aim of this study is to track the dynamic alterations in nutrient intake and digestion, rumen fermentation and plasma metabolic characteristics, and rumen bacterial community of Holstein finishing steers in response to three nutrient density diets as fattening phases advanced. A total of eighteen Holstein steers were randomly allocated into three nutrient density groups and steers in each group were fed under a three-phase fattening strategy, with nutrient density increased in each group when fattening phase advanced. Results showed that both fattening phase and dietary nutrient density significantly influenced the nutrient digestion, most of the rumen fermentation parameters, and part of bacteria at phylum and genus levels. Individually, dietary nutrient density affected the concentrations of plasma alanine aminotransferase and urea N, bacterial richness and evenness. All determined nutrient intake and plasma biochemical parameters, except for alanine aminotransferase and triglyceride, differed among fattening phases. Spearman correlation analysis revealed strong correlations between fiber intake and bacterial richness and evenness, rumen fermentation characteristics and certain bacteria. Moreover, *Patescibacteria* abundance was positively correlated with ambient temperature and plasma total protein. These results indicate that rumen fermentation and nutrient digestion were influenced by both dietary nutrient density and fattening phase, and these influences were regulated by certain rumen bacterial community and ruminal bacteria may be affected simultaneously by ambient temperature. This study may provide insights into diet optimization and potentially adaptive mechanism of rumen bacterial community in response to fattening phases and gradually climatic change.

## 1. Introduction

The rumen is of peculiarity to ruminants and harbors complex microbiota, including bacteria, protozoa, archaea, and fungi, which together play pivotal roles in transforming plant material into exploitable nutrients. Among these ruminal microorganisms, bacteria are the most diverse population and have been investigated intensively in recent years [1,2,3]. Many studies have revealed the correlations between bacterial community and host productivity [1,4]. Therefore, a better understanding of rumen bacterial community will be beneficial in improving the productivity of ruminants.

It is proverbial that both diet and age impact the composition of the rumen bacteria [3,5]. Numerous studies have revealed that cattle fed grain-based diet had lower bacterial diversity as compared to forage-rich diet and these two typical diets possessed distinct communities [3,6,7]. Jami et al. [5] characterized the dynamic bacterial compositions at five representative stages from birth to adulthood and observed convergent bacterial community as age advanced. Additionally, our previous studies in finishing steers revealed monthly temporal dynamics in composition of rumen bacteria even fed the same diet [8] and time-dependent alterations of fecal bacterial community in fattening stage [9]. However, information about the responses of ruminal bacteria to fattening diets in various nutrient densities, fattening stage, and their possible interaction is limited.

Ambient environment also influences bacterial composition and the dynamic trends of bacterial community [10]. The predominant microbiota was quite different in seasons and opposite variation trend of *Bacteroidetes* abundance was observed in summer and winter [10]. Previous report revealed that both *Succinivibrionaceae_UCG-002* and *Ruminobacter* abundances in rumen were positively associated with ambient temperature [8]. Yadav et al. [11] found that low ambient temperature reduced the mean retention time of ruminal contents, thus low digestibility of digesta may appear. Niu et al. [12] observed strong correlations between certain genera and apparent digestibility of fiber. However, the potential associations among ambient temperature, rumen bacteria, and nutrient digestibility are still limited. 

A typical beef cattle production system in China involves two or more fattening stages, with each stage lasts three or more months. Tracking the same animals across different fattening stages can provide the possibility to capture the variations of the bacterial composition and physiological or digestive characteristics under production conditions, and further provide decision-making for practical cattle production. Therefore, the aim of this study is to track the dynamics in nutrient intake and digestion, rumen fermentation and plasma metabolic characteristics, and rumen bacterial community in three nutrient density diets as fattening phases advanced, and further to characterize the potential relationships between them.

## 2. Materials and Methods 

### 2.1. Ethics Statement

Animal care and experimental procedures were approved by the Animal Care and Use Committee of China Agricultural University (Permit No. AW09059102-2).

### 2.2. Experiment Design and Sample Collection

Animal care and experimental design were according to our previous study [9]. Briefly, a total of eighteen Holstein steers, with average weight of 467 kg and average age of 14 months, were equally assigned to one of the three dietary treatments: high energy and high protein diet (H), moderate energy and moderate protein diet (C), and low energy and low protein diet (L). Steers in each treatment were fed with three-step strategy to meet or exceed the requirement of NRC [13], with the first three months named as phase 1, the second three months named as phase 2, and the last five months named phase 3. Dietary density in each dietary treatment increased as fattening phases advanced, which is commonly seen in practice in China. In addition, diets in each phase were designed to keep similar metabolizable energy to metabolizable protein ratio to minimize the possible effects due to protein balance. A detailed description of ingredients and nutrient composition is provided in Table 1. 

Feed intake and refusal were recorded daily, and the temperature and humidity were collected a quarter of an hour during the 334-day trail. Plasma samples were collected from the coccygeal vein into evacuated tubes with heparin sodium two hours before morning feeding on last day of each month, and then centrifuged at 4000 rpm/min for 20 min to obtain the plasma. Plasma samples were aliquoted into 1.5-mL centrifuge tubes and stored at −80 °C before determination by an automatic biochemical analyzer (Hitachi 7020; Hitachi Co., Tokyo, Japan).

Ruminal content was obtained three hours after morning feeding every month as described by Paz et al. [14], wherein both solid and liquid fractions were collected using esophageal tubing. Fecal samples were collected on three consecutive days every month, with sampling interval of six hours on each day. Feces from three days in each month of each steer were equally mixed into one sample for chemical analysis.

### 2.3. DNA Extraction and Volatile Fatty Acids (VFA) Determination

One hundred and twenty-six ruminal samples (42 samples from each fattening phase, with 4, 5, and 5 in H, C, and L diets, respectively) were extracted using a DNA Kit (OMEGA, Omega Bio-Tek, Norcross, GA, USA) as described by Qiu et al. [8]. Purity and quality of genomic DNA were checked on 1% agarose gels and quantified using a spectrophotometer (NanoDrop 2000 Technologies Inc., Wilmington, DE, USA).

Ruminal pH was immediately determined by a portable pH meter (Testo 205, Testo AG, Schwarzwald, Germany) after collecting digesta from rumen. Ruminal contents were centrifuged at 20,000× *g* for 20 min at 4 °C, and the supernatants were the original samples for subsequent VFA and ammonia nitrogen (NH_3_-N) determination. VFA measurement was according to our previous report [9]. Briefly, 800 μL separated ruminal fluid was mixed evenly with 200 μL internal standard solution (filling 25 g metaphosphoric acid and 217 μL 2-ethylbutyric acid with sterile water to 100 mL) and the determination was by means of a gas chromatograph (GC-2014 Shimadzu Corporation, Kyoto, Japan) with a 30-m capillary column (Rtx-Wax, 0.25 mm ID × 0.25 μm film, Restek, Evry, France) and nitrogen as the carrier gas. The oven program was well optimized as follows: initial 110 °C for 30 s, up to 120 °C at a constant rate of 10 °C/min, 120 °C hold for 4 min, and gradually rise to 150 °C in 3 min. The split ratio and flow rate were controlled at 20:1 and 2.5 mL/min, respectively. NH_3_-N was determined according to Broderick and Kang [15] using the phenol-sodium hypochlorite colorimetry.

### 2.4. PCR Amplification, Sequencing, and Analysis

The primers and PCR reaction system used in the current study were according to our previous report [8]. Briefly, the V3 to V4 was selected as the target hypervariable region, which was amplified with the following primers: 338F (5′-ACTCCTACGGGAGGCAGCAG-3′) and 806R (5′-GGACTACHVGGGTWTCTAAT-3′). A well-constructed 25-μL reaction system was performed as 12.5 μL of KAPA 2G Robust Hot Start Ready Mix, 2 μL equal amount of forward and reverse primers (5 μM), 5 μL of ruminal DNA, and 5.5 μL of sterile double distilled water. The well-mixed reaction was amplified under the following program: 95 °C for 5 min; followed by 32 cycles of 95 °C for 45 s, 55 °C for 50 s, and 72 °C for 45 s; and 72 °C for 10 min. Each sample was performed in triplicate, and PCR products were evaluated on 2% agarose gels and purified using an DNA Gel Extraction kit (AxyPrep, Axygen Biosciences, Union City, CA, USA). Paired-end sequences were obtained via the Illumina MiSeq platform (San Diego, CA, USA).

The raw data were analyzed using QIIME (version 1.9.1, https://qiime.org/). Paired end reads were merged by FLASH (version 1.2.11, http://ccb.jhu.edu/software/FLASH/). The length of the sequences was controlled at minimum of 260 bp and maximum of 500 bp. Sequences that contain any ambiguous base or chimera, with quality score of below 20 or mismatching to primer sequences were out of consideration. Filtered sequences were clustered into operational taxonomic units (OTUs) at a similarity level of 0.97 using UPARSE method (USEARCH v11.0.667, http://www.drive5.com/usearch/). OTUs across all samples were rarefied to the lowest sample depth (38,708 reads) based on the pseudo-random number generator of QIIME. Alpha diversity metrics were calculated using Mothur (version 1.39.5, Patrick Schloss, Ann Arbor, USA) [16]. The Ribosomal Database Project (RDP) classifier (http://sourceforge.net/projects/rdp-classifier/) was selected to taxonomy classifications by assigning against the SILVA bacterial alignment database version 132 [17]. Spearman correlation coefficients (r) and FDR corrected values (q) were calculated using the Psych packages version 1.8.12 to show correlations between ambient temperature, nutrient intake and apparent digestibility, plasma biochemical parameters, rumen fermentation characteristics, and bacterial composition. The graph was finished by means of GraphPad Prism (version 8.0.2, GraphPad Software, Inc., San Diego, CA, USA). 

The raw sequences involved in this study were deposited in the Sequence Read Archive (SRA) of NCBI, and the SRA accession number is PRJNA 573499. It should be noted especially that 30 ruminal samples from phase 1 had been taken to explore the sampling frequency regarding rumen bacterial community composition in our previous study [8], but the analysis methods and purposes were different from the current study.

### 2.5. Chemical Analysis and Apparent Digestibility Calculation

The dry matter (DM, method 934.01), crude protein (CP, method 990.03), ether extract (EE, method 920.39) and ash (method 942.05) of feeds, orts and feces were analyzed according to the methods of the Association of Official Analytical Chemists [18]. Neutral detergent fiber (NDF) and acid detergent fiber (ADF) were measured according to Van Soest et al. [19], with the former assayed with alpha amylase. Acid-insoluble ash was selected as a natural marker to estimate the total feces, and the apparent digestibility of nutrient was calculated using the method described by Niu et al. [12].

### 2.6. Statistical Analysis

The average daily temperature-humidity index (THI) was calculated according to Vitali et al. (2009), which is expressed as (1.8 × T + 32) – (0.55 – 0.55 × H/100) × [(1.8 × T + 32) – 58], where T denotes temperature (°C) and H indicates relative humidity (%); THI greater than 74 implied that cattle suffered from heat stress [20]. All three samples from the same steer in each fattening phase were averaged as one value to show a better presentation, because our previous study [8] showed that ruminal bacteria were in dynamic variation before an adaptation period of three months. Therefore, each fattening phase had 18 average values for each parameter, except for the bacterial community. All averaged data were analyzed as a repeated measures design in Mixed Models procedure of SPSS (version 21, IBM Corporation, Armonk, New York, United States) with first-order autoregressive covariance structure after comparing compound symmetry, first-order ante dependence and unstructured covariance structure based on the minimum Akaike information criterion (AIC) values. The statistical model used for this analysis is expressed as follows: *y*_ijt_ = μ + α_i_ + γ_t_ *+ c*_j(i)_ + (αγ)*_it_* + *e_ijt_*, where *y*_ijt_ is the parameter measured at fattening phase t on the *j*th cattle allotted to the *i*th diet, μ is the overall mean, α_i_ is the *i*th fixed diet effect, γ_t_ is the fixed *t*th fattening phase effect, *c*_j(i)_ is the random effect of the *j*th cattle within the *i*th diet, (αγ)*_it_* is the fixed interaction effect between diet and fattening phase, and *e_ijt_* is the random error in the *j*th cattle allotted to the *i*th diet in fattening phase *t*. Multiple comparison was conducted using Tukey tests and significance was declared at 0.05 (*p* < 0.05).

## 3. Results

### 3.1. Temperature-Humidity Index, Nutrient Intake, and Apparent Digestibility

The average semimonthly temperature and humidity are presented in Figure S1. Days with THI over 74 were 0, 4, and 69 in phase 1, phase 2 and phase 3, respectively. Additionally, the proportion of days with THI over 74 to all days (69/154) in last phase was 44.81%. The nutrient intake and apparent digestibility of nutrient are shown in Table 2. All determined nutrient intake differed significantly (*p* < 0.001) in three fattening phases, with the highest in phase 2, followed by phase 1 and phase 3. The intakes of CP and EE increased as dietary nutrient density increased, whereas intakes of NDF and ADF decreased as dietary nutrient density increased (*p* < 0.05), with no differences (*p* > 0.10) in intakes of DM and OM due to dietary treatments. No interaction effects (*p* > 0.10) were found in nutrient intakes between fattening phase and dietary treatment. 

Both fattening phase and dietary treatment influenced the apparent digestibility of measured nutrients. The apparent digestibility of DM, OM, CP, and EE were higher (*p* < 0.001) in phase 3 as compared to phase 1 and phase 2, whereas apparent digestibility of NDF and ADF were higher (*p* < 0.001) in phase 1 when compared with phase 2 and phase 3. The apparent digestibility of DM, OM, CP, and EE were higher (*p* < 0.05) in steers fed H diet than steers fed C and L diets, and opposite results were observed in the apparent digestibility of NDF and ADF. Interaction effects were observed in the apparent digestibility of ADF and EE between fattening phase and dietary treatment.

### 3.2. Plasma Metabolic Parameters

The plasma metabolic parameters are summarized in Table 3. Fattening phase significantly influenced (*p* < 0.05) the detected metabolic parameters, except for alanine aminotransferase (ALT) and triglyceride. The concentration of ALT was higher (*p* = 0.005) in steers fed L diet than that in steers fed H and C diets, and opposite result was observed in concentration of urea N (*p* < 0.001). Interaction effects were observed in concentrations of glucose, triglyceride, and urea N between fattening phase and dietary treatment.

### 3.3. Rumen Fermentation Characteristics 

The rumen fermentation characteristics are shown in Table 4. Rumen pH, VFA, and total VFA (TVFA) significantly differed (*p* < 0.05) in three fattening phases, whereas no differences (*p* > 0.05) were observed in concentration of NH_3_-N and ratio of acetate to propionate (A/P). Dietary nutrient density altered (*p* < 0.05) most of the rumen fermentation profiles, except for concentrations of NH_3_-N and TVFA. Interaction effects, except for concentrations of NH_3_-N and acetate, were observed between fattening phase and dietary treatment.

### 3.4. Rumen Bacterial Community

A total of 16, 340, 654 sequencing reads were obtained after filtering low-quality data, with 99.99% of high-quality sequences between the lengths of 380 bp and 440 bp. After clustering high-quality sequences, an average of 1446 OTUs was obtained for each sample. The converged and slow-growing Shannon-Wiener curves (Figure S2) indicated that the sequencing depth in the current study was adequate to evaluate the vast composition of ruminal bacteria.

The estimation of alpha diversity is listed in Table 5. No differences (*p* > 0.05) in richness and evenness were observed among fattening phases. Chao 1, observed species, PD whole tree, and Shannon index increased as dietary nutrient density decreased. An interaction was observed in Shannon index between fattening phase and dietary treatment.

As is shown in Table 6, ten phyla were observed with relative abundance over 0.5%, and *Bacteroidetes* and *Firmicutes* dominated the most two. Fattening phase affected (*p* < 0.05) abundances of *Bacteroidetes*, *Firmicutes*, *Proteobacteria*, *Fibrobacteres,* and *Patescibacteria*, whereas dietary treatment had significant effects (*p* < 0.05) on eight out of ten phyla, except for *Bacteroidetes* and *Actinobacteria*. Besides, no interactions (*p* > 0.05) were observed between fattening phase and dietary treatment regarding phyla with relative abundance greater than 0.5%.

The taxonomic analysis with average abundance over 0.5% at the genus level is shown in Table 7. Twenty-six genera were observed with relative abundance greater than 0.5%, with *Prevotella* predominated 32.78%, 29.29%, and 26.86% in H, C, and L diets, respectively. Eight and ten out of twelve with relative abundance over 1% were affected (*p* < 0.05) by fattening phase and dietary treatment, respectively. Of these, no interactions (*p* > 0.05) were observed between fattening phase and dietary treatment. However, interactions were found in *Veillonellaceae UCG-001*, *Prevotellaceae NK3B31 group*, and *Saccharofermentans* between fattening phase and dietary treatment, whose relative abundances were below 1%.

### 3.5. Correlation Analysis

**Figure 1:**
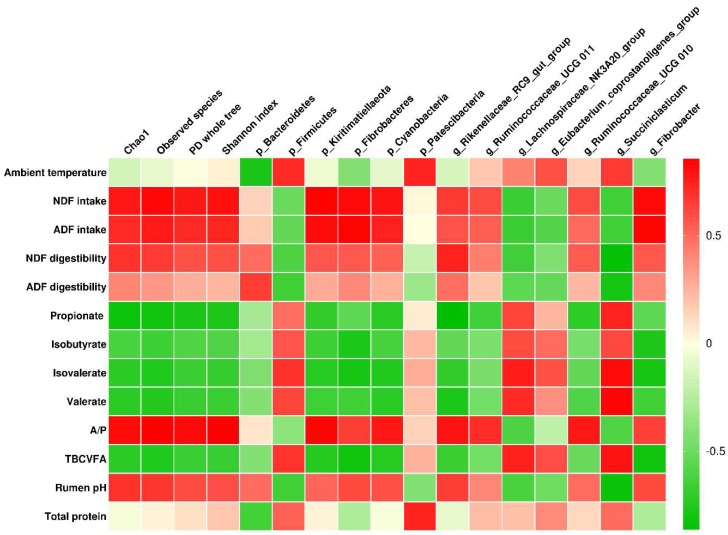
Correlations between ambient temperature, physiological and digestive characteristics, and bacterial abundances of Holstein finishing steers fed three nutrient density diets. Only significant correlations (r > 0.70 or r < −0.70 and q-value < 0.05) and bacteria abundances greater than 0.5% are shown. The colors indicate positive (red, closer to 1) or negative (green, closer to −1) correlations between investigated characteristics and bacterial abundances. A/P, acetate to propionate ratio; TBCVFA, total branched-chain volatile fatty acids.

Correlations between ambient temperature, nutrient intake and apparent digestibility, plasma metabolic and rumen fermentation characteristics, and rumen bacterial composition are shown in Figure 1. Ambient temperature was positively associated with the phyla *Firmicutes* and *Patescibacteria* (r > 0.7 and q < 0.01), and it also correlated negatively with the phylum *Bacteroidetes* (r = −0.770 and q < 0.001). The estimated alpha diversity (both richness and evenness) was negatively associated with propionate (r < −0.7 and q < 0.01) and positively associated with intakes of NDF and ADF and A/P (r > 0.7 and q < 0.01); richness (Chao 1 and observed species) correlated negatively with isovalerate, valerate, and total branched-chain VFA (BCVFA) (r < −0.7 and q < 0.01). 

The intakes of NDF and ADF were positively correlated with phyla *Kiritimatiellaeota*, *Cyanobacteria,* and *Fibrobacteres* (r > 0.7 and q < 0.01). The apparent digestibility of NDF and ADF and rumen pH were negatively associated with the genus *Succiniclasticum* (r < −0.7 and q < 0.01), whereas the relative abundance of *Succiniclasticum* was positively correlated with propionate, isovalerate, valerate, and total BCVFA (r > 0.7 and q < 0.01). The plasma total protein correlated positively with the phylum *Patescibacteria* (r = 0.734 and q < 0.01). The concentration of propionate was negatively correlated with the genus *Rikenellaceae RC9 gut group* (r = −0.865 and q < 0.01). The A/P was positively associated with the relative abundance of *Kiritimatiellaeota* (r = 0.837 and q < 0.01).

## 4. Discussion

### 4.1. Digestive and Physiological Characteristics

It is generally accepted that nutrient intake increases as animals grow, so as to meet the requirement of maintenance and weight gain [13]. However, nutrient intake is affected by both feed intake and dietary nutrient density. In this study, nutrient intake decreased in phase 3 because of low feed intake because steers suffered from heat stress at over 40% of time in phase 3. Besides, nutrient intake increased as dietary nutrient density improved as a result of similar feed intake in each phase (Table 2).

Steers in phase 3 had higher apparent digestibility of DM, OM, CP, and EE, which is similar to the reports of Yadav et al. [11], and these differences could be due to longer retention time of digesta in phase 3 because previous study revealed digesta retention time increased as ambient temperature increased when temperature was below 35 °C [11]. However, this effect was quite different in the apparent digestibility of NDF and ADF. Previous study reported that the activity of cellulolytic bacteria was partly inhibited by low ruminal pH and further reduced the digestibility of NDF and ADF [21]. In this study, ruminal pH values were lower in phase 3 as compared to that in phase 1 and phase 2, thereby a lower digestibility of NDF and ADF was observed. Steers fed H diet improved the apparent digestibility of DM, OM, and EE, which could be attributed to the high starch content in H diet, a digestible nutrient for ruminants. The higher apparent digestibility of CP in H diet was similar to the result of Menezes et al. [22], who suggested a positive association between apparent digestibility of CP and protein level. Dietary nutrient density decreased the apparent digestibility of NDF and ADF, which is in accordance with the results of Tjardes et al. [23], who suggested that high-fiber diet increased the apparent total-tract digestibility of both NDF and ADF. These differences could be explained by the longer rumen retention time and more cellulolytic bacteria induced by higher content of fiber [21]. 

Previous study [24] reported that most blood biochemical characteristics changed with ages. In this study, albumin, cholesterol, glucose, total protein and urea N differed among fattening phases because of generally physiological reasons, which are consistent with the reports of Mohri et al. [24]. The urea N is the main end-product of protein hydrolysis and amino acid metabolism [25], and its concentration has shown to be positively associated with intake of CP [12]. Therefore, it was reasonable that plasma urea N increased as dietary nutrient density increased as a result of increased CP intake. 

The ruminal pH is a vital ruminal characteristic because of its direct influence on microbial growth, and the adaptable pH for microbiota ranges from 5.5 to 7.0 [26]. In the current study, the ruminal pH values were in the tolerable range and decreased as dietary nutrient density improved and fattening phase advanced. High-density diet decreased the saliva production and chewing activities [27], and subsequently diminished the buffering capacity of suddenly increased VFA because of the high intake of fermentable carbohydrates. In addition, these differences among diets were more obvious in the last fattening phase, indicating that long-term stimulation would enlarge the effect of diets on ruminal pH, as seen by the interaction effect between fattening phase and diet regarding ruminal pH values. Generally, structural carbohydrates are mainly fermented into acetate and nonstructural carbohydrates produce more propionate [13], which was also mirrored in L and H diets in the present study, respectively. The A/P increased as dietary nutrient density decreased in the current study, which is in accordance with the fact that the A/P increased as the forage to concentrate ratio increased [28]. BCVFA usually derives from degradation of crude protein and has been used as an indicator of ruminal protein fermentation [29]. Higher concentrations of isobutyrate and isovalerate, as well as total BCVFA, were observed in H and C diets because of higher CP intake in these two diets. Our results showed high concentration of butyrate in H diet, which was similar to the report of Xia et al. [28] wherein bulls fed diet with high concentrate to forage ratio yielded high concentration of butyrate. Previous study [30] reported an elevated valerate concentration as the proportion of dietary concentrate increased. In this study, similar results in valerate concentration were obtained in terms of both diet and fattening phase, because higher dietary nutrient density, as well as later fattening phases, contained more fermentable nonstructural carbohydrates.

### 4.2. Rumen Bacterial Community Profiles

Alpha diversity metrics are used to estimate the species richness and evenness in a certain sample or a single community [31]. In this study, rumen samples from steers fed C and L diets showed higher diversity as compared to H diet, which is in line with many reports [3,6,7] in which grain-based diet decreased alpha diversity. These differences may be explained by the well-established theory that ruminal pH has a large impact on the rumen bacterial diversity [32].

*Bacteroidetes* and *Firmicutes* dominated about 90% of the bacterial composition, with 62.88% and 27.15%, respectively. The higher abundance of *Firmicutes* in steers fed H and C diets was due to lower ruminal pH in these two diets because previous study reported that lower ruminal pH increased the proportion of *Firmicutes* in bacterial composition [32]. *Proteobacteria* plays an important role in the rumen metabolism despite of the relative low abundance, and was frequently observed in starch-rich diet [3,33]. However, present study showed the opposite result wherein high abundance of *Proteobacteria* was observed in fiber-rich diet. It is tempting to assume that certain species in the phylum *Proteobacteria* may also actively take part in the digestion of fiber, but further studies are needed to confirm this assumption and certain species. *Kiritimatiellaeota*, a novel phylum previously assigned to the *Verrucomicrobia* subdivision 5, occupied niches mainly characterized by anaerobic environment [34]. The relative abundance of *Kiritimatiellaeota* increased as dietary nutrient density decreased, indicating that this phylum may be involved in fiber degradation. *Fibrobacteres*, as well as *Fibrobacter*, is well-known for its vital role in degrading cellulose, and they are commonly detected in fiber-rich diet [3]. Therefore, it is obvious to expect a decreasing abundance as dietary nutrient density increased in the present study.

Significant difference in *Prevotella* abundance was observed between steers fed H and L diets because of its function in starch fermenting and protein metabolism [35]. Similar result was observed in relative abundance of *Prevotellaceae UCG-001*, a genus belongs to the same family *Prevotellaceae*. However, *Prevotellaceae UCG-003* showed the opposite results, which increased as dietary nutrient density decreased. These results regarding *Prevotellaceae UCG-003* were similar to the report of Liu et al. [6] wherein higher proportion of *Prevotellaceae UCG-003* was found in forage-based feeding group, indicating that different genera in the same family may involve in diverse metabolic function. *Rikenellaceae RC9 gut group* was previously regarded to be involved in the degradation of carbohydrates [36] and recent study in Holstein bulls has revealed the negative association between propionate concentration and *Rikenellaceae RC9 gut group* abundance [33]. In this study, the relative abundance of *Rikenellaceae RC9 gut group* decreased as dietary nutrient density increased which resembled to our previous research in feces [9], indicating that this genus may also be involved in degrading fiber in the rumen. The present study emerged a cumulative increase in *Succiniclasticum* abundance as dietary nutrient density increased, which is similar to previous report wherein high proportion of this genus was found in high-grain feeding pattern [6]. Previous study observed that Yak fed with more concentrate harbored higher abundance of *Ruminococcaceae NK4A214 group* and *Ruminococcus 2* [6], which applied equally to Holstein steers fed with high density diet in the present study, indicating these two genera had the potential to degrade nonfibrous material. *Ruminococcaceae UCG-011*, a genus belonging to the family *Ruminococcaceae*, has been reported to be cellulose utilizer in rumen of beef cattle and higher proportion of this genus was observed in forage-based diet as compared to grain-rich diet [6], this is similar to our findings wherein relative abundance of *Ruminococcaceae UCG-011* increased as the dietary nutrient density decreased.

### 4.3. Correlations between Ambient Temperature, Physiological and Digestive Characteristics, and Ruminal Bacterial Community

In this study, correlation analysis revealed the relationships between ambient temperature and rumen bacterial abundances. The superphylum *Patescibacteria* was commonly detected in anoxic environments and potentially involves anaerobic fermentative metabolisms [37]. Therefore, it is possible that *Patescibacteria* did exist in rumen because of anaerobic environment in rumen. In this study, *Patescibacteria* abundance was positively correlated with ambient temperature, indicating that *Patescibacteria* abundance in rumen could be influenced by ambient temperature, which could also be seen from higher abundance of this superphylum in phase 3 than in phase 1 and phase 2 (Table 6). Previous study reported opposite variation trend in relative abundances of *Bacteroidetes* and *Firmicutes* from winter to summer [10], and similar results were also observed in the present study, with negative correlation between ambient temperature and *Bacteroidetes* and positive correlation between ambient temperature and *Firmicutes*. Interestingly, one of the plasma metabolic parameters, total protein, was observed to be positively associated with the abundance of the superphylum *Patescibacteria*. The concentration of total protein depends on the absorption of globulin and albumin [38]. The present results indicate that *Patescibacteria* may indirectly accelerate the absorption of total protein. To our knowledge, this is the first time to observe association between plasma total protein concentration and *Patescibacteria* abundance.

The intake of fiber (NDF and ADF) correlated positively with alpha diversity indexes. This could be explained from two aspects. On the one hand, more intake of fiber indicates greater degradable fiber and fiber digestibility (Table 2), and greater fiber degradation indicates greater substrate and metabolite releases [39], thus the microbial populations have greater concentration of substrates for their living. On the other hand, greater intake of fiber carbohydrates keeps optimal ruminal acidity by stimulating rumination and saliva production [27], thus provides a favorable environment for the microbiota to grow. In addition, positive correlations were observed between NDF and ADF intake and *Fibrobacteres*, *Kiritimatiellaeota,* and *Cyanobacteria*. Among these phyla, *Fibrobacteres* is a typically cellulolytic phylum; *Kiritimatiellaeota* is potentially involved in fiber degradation; and *Cyanobacteria* was found to participate in degrading plant polysaccharides [1]. *Succiniclasticum* plays vital roles in degrading starch and transforming succinate into propionate [40], which explained the positive association between *Succiniclasticum* and propionate concentration. As described previously, propionate is the main product fermenting from nonstructural carbohydrates, and this type of carbohydrates are fermented rapidly and may restrict fiber fermentation [27], resulting in quite low ruminal pH which is unsuitable for microbial growth. Therefore, it is reasonable to see negative correlations between propionate concentration and bacterial diversity, as well as negative correlations between bacterial diversity and digestibility of NDF and ADF, and between bacterial diversity and rumen pH value. Moreover, propionate concentration correlated negatively with *Rikenellaceae RC9 gut group* abundance, which is very similar to the report of Wang et al. [33], indicating potential function of this genus in impeding propionate fermentation. A/P represents the ratio of structural carbohydrates to nonstructural carbohydrates in diet because of their main end product are acetate and propionate, respectively [13]. As described above, nonstructural carbohydrates generally ferment rapidly and result in low bacterial diversity due to lower ruminal pH, thus positive associations were observed between A/P and alpha diversity indexes. A/P was also positively correlated with *Kiritimatiellaeota*, proving again the involvement of this phylum in degradation of structural carbohydrates. Valerate is one of the products resulting from the degradation of branched amino acids and itself serves as an important precursor for synthesizing odd branched-chain fatty acids [6]. It has also been suggested that odd branched-chain fatty acids could be synthesized by elongating propionate [41]. Hence, it is reasonable to observe positive correlation between *Succiniclasticum* abundance and concentrations of propionate, isovalerate, valerate, and total BCVFA due to vital roles of this genus in fermenting succinate to propionate and degradation of protein [12,40].

## 5. Conclusions

In summary, both dietary nutrient density and fattening phase significantly impacted nutrient intake and digestion, rumen fermentation characteristics and rumen bacterial community. Besides, dietary nutrient density affected concentrations of ALT and urea N in plasma, rumen bacterial richness and evenness, while fattening phase influenced most of metabolic characteristics, except for ALT and triglyceride. The interaction effects between dietary treatment and fattening phase throughout the investigation indicate that continuous stimulations deepen the effect of dietary nutrient density on physiological and digestive characteristics, and bacterial community. Furthermore, correlation analysis indicates that diet and fattening phase, as well as ambient temperature, play crucial role in regulating digestive and physiological metabolisms and rumen bacteria.

## Figures and Tables

**Table 1:** Ingredients and nutrient compositions of the diets.

Item ^1^	Phase 1			Phase 2			Phase 3		
	H	C	L	H	C	L	H	C	L
Ingredients, g/kg of DM									
Corn grain	529	414	299	588	468	348	620	501	382
Wheat grain	73.2	57.3	41.4	71.7	57.1	42.5	87.8	70.9	54.0
Soybean meal	94.1	73.7	53.2	93.7	74.6	55.5	89.7	72.5	55.3
*Leymus chinensis*	282	438	594	216	376	536	169	329	489
Limestone	7.20	5.60	4.10	7.80	6.20	4.60	8.30	6.70	5.10
NaHCO_3_	0.00	0.00	0.00	7.80	6.20	4.60	8.30	6.70	5.10
NaCl	7.10	5.60	4.00	7.80	6.20	4.60	8.30	6.70	5.10
Premix ^2^	7.20	5.60	4.10	7.80	6.20	4.60	8.30	6.70	5.10
Nutrient composition ^3^, g/kg of DM									
ME, Mcal/kg	2.71	2.53	2.35	2.77	2.58	2.40	2.82	2.64	2.46
MP, g/kg	82.9	73.9	64.8	83.9	75.2	66.4	84.3	76.0	67.7
ME/MP, Mcal/g	0.033	0.034	0.036	0.033	0.034	0.036	0.034	0.035	0.036
NDF	277	371	464	236	332	428	208	304	400
ADF	146	202	258	121	179	237	104	162	219
Ether extract	34.6	32.5	30.5	35.6	33.4	31.3	36.1	34.0	31.9
Main fatty acid profile ^4^ (mg/g feed, DM)									
C16:0	3.87	3.53	3.19	3.33	3.12	2.92	4.06	3.73	3.39
C18:1n9c	3.64	2.92	2.21	3.25	2.66	2.07	4.25	3.50	2.75
C18:2n6	4.27	3.47	2.67	3.37	2.80	2.24	3.55	2.98	2.41
SFA	6.01	5.61	5.20	4.85	4.71	4.56	5.88	5.55	5.21
UFA	8.75	7.53	6.31	7.33	6.48	5.62	8.48	7.46	6.43

^1^ H = high energy and high protein diet group; C = moderate energy and moderate protein diet group; L = low energy and low protein diet group. The ingredients and nutrient composition were in accordance with our previous study (Qiu, et al. 2019). ^2^ Manufactured by Tangshan Mahanen Feed Co., Ltd., Tangshan, China; premix provided the following per kg of dry matter (DM): 5000 IU of vitamin A, 3000 IU of vitamin D3, 45 mg of vitamin E, 60 mg of Fe, 63 mg of Zn, 99 mg of Mn, 200 mg of Cu, 0.5 mg of Se, 1.1 mg of I, 0.45 mg of Co, 877 g of rice bran. ^3^ DM, dry matter; ME, metabolizable energy; MP, metabolizable protein, predicted according to NRC (2016) using average values for RUP and RDP; NDF, neutral detergent fiber; ADF, acid detergent fiber. ^4^ SFA, saturated fatty acids; UFA, unsaturated fatty acids.

**Table 2:** Effects of dietary nutrient density on nutrient intake and apparent digestibility in the whole fattening phases of Holstein steers.

Item ^1^	Phase 1			Phase 2			Phase 3			SEM ^2^	*p*-Value ^3^		
	H	C	L	H	C	L	H	C	L		Phase	Diet	P × D
Nutrient intake, kg/d													
Dry matter	10.7	11.3	10.6	13.5	13.4	12.9	11.9	12.5	12.8	0.41	<0.001	0.491	0.401
OM	10.2	10.7	10.0	12.4	12.4	12.0	11.3	11.8	12.1	0.39	<0.001	0.489	0.445
Crude protein	1.41	1.33	1.10	1.69	1.47	1.21	1.41	1.30	1.16	0.05	<0.001	<0.001	0.168
Ether extract	0.40	0.39	0.34	0.51	0.47	0.41	0.45	0.44	0.42	0.02	<0.001	<0.001	0.448
NDF	2.92	4.11	4.79	3.03	4.28	5.29	2.16	3.31	4.44	0.11	<0.001	<0.001	0.231
ADF	1.50	2.20	2.63	1.53	2.28	2.90	1.00	1.63	2.25	0.06	<0.001	<0.001	0.242
Apparent digestibility of nutrient, %													
Dry matter	69.8	68.6	68.2	71.0	65.1	66.5	78.9	75.5	74.1	1.42	<0.001	0.004	0.477
OM	71.4	70.8	70.5	72.4	66.8	68.7	80.5	77.5	76.5	1.45	<0.001	0.019	0.469
Crude protein	71.5	67.8	67.9	70.4	62.6	61.6	76.7	72.2	69.5	1.79	<0.001	<0.001	0.574
Ether extract	71.8 ^bcd^	69.9 ^cd^	64.0 ^d^	69.7 ^cd^	66.4 ^d^	71.5 ^cd^	84.9 ^a^	79.7 ^ab^	75.5 ^bc^	1.73	<0.001	0.003	0.006
NDF	64.7	68.7	74.3	55.0	60.8	67.5	59.4	64.2	66.3	1.65	<0.001	<0.001	0.486
ADF	65.2 ^bc^	68.2 ^ab^	73.6 ^a^	55.2 ^e^	58.1 ^de^	62.4 ^bcd^	56.0 ^de^	61.0 ^cde^	57.3 ^de^	1.46	<0.001	<0.001	0.023

^1^ OM, organic matter; NDF, neutral detergent fiber; ADF, acid detergent fiber. Phase 1 indicates fattening phase 1 (1–3 month), phase 2 indicates fattening phase 2 (4–6 month), and phase 3 indicates fattening phase 3 (7–11 month). H = high energy and high protein diet, C = moderate energy and moderate protein diet, and L = low energy and low protein diet. ^2^ SEM, standard error of means. ^3^ P × D indicates interaction effect between fattening phase and dietary treatment, lowercase letters are marked only when the interaction effect was significant.

**Table 3:** Effects of dietary nutrient density on plasma biochemical parameters in the whole fattening phases of Holstein steers.

Item ^1^	Phase 1			Phase 2			Phase 3			SEM ^2^	*p* – Value ^3^		
	H	C	L	H	C	L	H	C	L		Phase	Diet	P × D
Albumin, g/L	34.03	33.70	32.89	33.70	34.62	33.98	35.85	34.59	35.26	0.44	0.001	0.476	0.139
ALT, U/L	20.6	20.8	24.1	20.2	24.4	25.8	23.5	19.7	25.7	1.19	0.232	0.005	0.091
AST, U/L	47.2	45.8	41.9	50.1	53.0	47.0	46.6	48.6	47.6	3.24	0.093	0.206	0.253
AST/ALT	2.36	2.24	1.68	2.49	2.21	1.81	2.16	2.55	1.86	0.21	0.621	0.075	0.208
Cholesterol, mmol/L	2.75	2.84	2.46	2.89	3.18	2.75	2.28	2.38	2.48	0.12	<0.001	0.211	0.072
Glucose, mmol/L	4.27 ^a^	4.13 ^ab^	3.95 ^abc^	3.74 ^bcd^	3.78 ^bcd^	3.49 ^d^	3.63 ^cd^	3.50 ^d^	3.86 ^bcd^	0.09	<0.001	0.369	0.007
Total protein, g/L	58.6	56.2	53.7	58.7	61.4	60.2	62.5	65.8	68.0	1.77	<0.001	0.701	0.116
Triglyceride, mmol/L	0.20 ^b^	0.22 ^ab^	0.29 ^a^	0.21 ^ab^	0.24 ^ab^	0.24 ^ab^	0.24 ^ab^	0.23 ^ab^	0.24 ^ab^	0.02	0.663	0.002	0.018
Urea N, mmol/L	4.07 ^a^	3.52 ^abc^	2.27 ^d^	3.77 ^ab^	3.61 ^abc^	2.59 ^d^	2.82 ^cd^	3.04 ^bcd^	2.78 ^cd^	0.18	0.008	<0.001	0.001

^1^ ALT, alanine aminotransferase; AST, aspartate aminotransferase; AST/ALT denotes AST to ALT ratio. Phase 1 indicates fattening phase 1 (1-–3 month), phase 2 indicates fattening phase 2 (4–6 month), and phase 3 indicates fattening phase 3 (7–11 month). H = high energy and high protein diet, C = moderate energy and moderate protein diet and L = low energy and low protein diet. ^2^ SEM, standard error of means. ^3^ P × D indicates interaction effect between fattening phase and dietary treatment, lowercase letters are marked only when the interaction effect was significant.

**Table 4:** Effects of dietary nutrient density on rumen fermentation characteristics in the whole fattening phases of Holstein steers.

Item ^1^	Phase 1			Phase 2			Phase 3			SEM ^2^	*p* – Value ^3^		
	H	C	L	H	C	L	H	C	L		Phase	Diet	P × D
NH_3_-N, mg/dL	4.41	3.66	2.56	4.65	4.23	3.04	5.01	4.31	3.16	1.156	0.679	0.313	0.998
Rumen pH	6.56 ^abc^	6.65 ^ab^	6.78 ^a^	6.26 ^de^	6.33 ^cde^	6.71 ^ab^	5.86 ^f^	6.25 ^e^	6.52 ^bcd^	0.054	<0.001	<0.001	0.002
Acetate, mM	39.1	46.5	48.3	46.8	53.2	56.5	47.0	50.8	56.1	1.570	<0.001	<0.001	0.620
Propionate, mM	13.5 ^b^	12.0 ^b^	10.3 ^b^	18.8 ^a^	13.7 ^b^	12.1 ^b^	21.0 ^a^	13.8 ^b^	10.7 ^b^	0.738	<0.001	<0.001	<0.001
Isobutyrate, mM	0.64 ^bc^	0.65 ^bc^	0.52 ^d^	0.62 ^cd^	0.66 ^bc^	0.52 ^d^	0.81 ^a^	0.74 ^ab^	0.57 ^cd^	0.022	<0.001	<0.001	0.013
Butyrate, mM	7.23 ^cde^	6.62 ^de^	6.29 ^e^	9.03 ^abc^	10.0 ^a^	8.20 ^abcd^	9.67 ^ab^	8.90 ^abc^	7.79 ^bcde^	0.401	<0.001	0.035	0.030
Isovalerate, mM	0.92 ^cde^	0.88 ^cde^	0.60 ^e^	1.16 ^c^	1.09 ^cd^	0.62 ^e^	2.17 ^a^	1.67 ^b^	0.78 ^de^	0.076	<0.001	<0.001	<0.001
Valerate, mM	0.78 ^cde^	0.68 ^ef^	0.58 ^f^	0.98 ^b^	0.85 ^bcd^	0.70 ^def^	1.40 ^a^	0.90 ^bc^	0.66 ^ef^	0.033	<0.001	<0.001	<0.001
TVFA, mM	62.2 ^b^	67.3 ^b^	66.7 ^b^	77.4 ^a^	79.6 ^a^	78.6 ^a^	82.1 ^a^	76.8 ^a^	76.5 ^a^	1.861	<0.001	0.937	0.008
A/P	2.98 ^de^	3.92 ^bc^	4.71 ^ab^	2.58 ^e^	3.93 ^bc^	4.67 ^ab^	2.41 ^e^	3.81 ^cd^	5.38 ^a^	0.171	0.380	<0.001	0.007
TBCVFA, mM	1.56 ^cde^	1.53 ^cdef^	1.12 ^f^	1.78 ^c^	1.74 ^cd^	1.14 ^ef^	2.98 ^a^	2.41 ^b^	1.35 ^def^	0.087	<0.001	<0.001	<0.001

^1^ NH_3_-N, ammonia nitrogen; TVFA, total volatile fatty acids; A/P, acetate to propionate ratio; TBCVFA, total branched-chain volatile fatty acids, equal to the sum of isobutyrate and isovalerate. Phase 1 indicates fattening phase 1 (1–3 month), phase 2 indicates fattening phase 2 (4–6 month), and phase 3 indicates fattening phase 3 (7–11 month). H = high energy and high protein diet, C = moderate energy and moderate protein diet, and L = low energy and low protein diet. ^2^ SEM, standard error of means. ^3^ P × D indicates interaction effect between fattening phase and dietary treatment, lowercase letters are marked only when the interaction effect was significant.

**Table 5:** Effects of dietary nutrient density on alpha diversity metrics in the whole fattening phases of Holstein steers.

Item ^1^	Phase 1			Phase 2			Phase 3			SEM ^2^	*p –* Value ^3^		
	H	C	L	H	C	L	H	C	L		Phase	Diet	P × D
Chao1	1852	2221	2407	1685	2116	2288	1601	1970	2425	90.78	0.053	<0.001	0.265
Observed species	1195	1440	1586	1112	1428	1567	1049	1305	1660	53.80	0.179	<0.001	0.064
PD whole tree	100.0	117.4	124.8	100.1	117.6	123.1	96.25	110.6	132.5	3.654	0.929	<0.001	0.093
Shannon index	7.456 ^d^	8.084 ^bc^	8.323 ^abc^	7.385 ^d^	8.167 ^bc^	8.445 ^ab^	7.364 ^d^	7.858 ^cd^	8.718 ^a^	0.113	0.855	<0.001	0.029

^1^ PD, phylogenetic diversity. Phase 1 indicates fattening phase 1 (1–3 month), phase 2 indicates fattening phase 2 (4–6 month), and phase 3 indicates fattening phase 3 (7–11 month). H = high energy and high protein diet, C = moderate energy and moderate protein diet, and L = low energy and low protein diet. ^2^ SEM, standard error of means. ^3^ P × D indicates interaction effect between fattening phase and dietary treatment, lowercase letters are marked only when the interaction effect was significant.

**Table 6:** Effects of dietary nutrient density on rumen bacterial composition at phylum level (relative abundance > 0.5%) in the whole fattening phases of Holstein steers.

Phylum Name ^1^	Phase 1			Phase 2			Phase 3			SEM ^2^	*p* - Value ^3^		
	H	C	L	H	C	L	H	C	L		Phase	Diet	P × D
*Bacteroidetes*	72.15	66.52	67.66	61.21	61.14	63.55	56.70	58.49	58.82	1.73	<0.001	0.482	0.269
*Firmicutes*	21.48	25.08	21.07	30.15	26.64	21.36	37.18	33.62	29.25	1.60	<0.001	0.001	0.135
*Proteobacteria*	1.75	2.58	2.46	2.40	4.45	3.62	1.24	2.14	3.00	0.54	0.022	0.008	0.579
*Kiritimatiellaeota*	0.48	1.17	1.90	0.49	1.30	3.49	0.46	0.79	2.12	0.32	0.096	<0.001	0.187
*Fibrobacteres*	0.86	1.12	2.39	1.04	1.51	3.01	0.38	0.45	1.27	0.20	<0.001	<0.001	0.122
*Spirochaetes*	0.64	0.85	1.07	0.79	0.96	1.27	0.80	0.66	0.90	0.12	0.101	0.037	0.431
*Cyanobacteria*	0.49	0.58	1.33	0.49	0.85	1.24	0.23	0.52	1.47	0.15	0.604	<0.001	0.362
*Patescibacteria*	0.38	0.58	0.53	0.33	0.80	0.64	0.98	1.09	0.90	0.08	<0.001	0.008	0.272
*Actinobacteria*	0.80	0.25	0.20	0.96	0.64	0.30	0.68	0.80	0.41	0.24	0.349	0.172	0.557
*Tenericutes*	0.41	0.66	0.59	0.44	0.66	0.52	0.28	0.42	0.72	0.08	0.426	0.013	0.078

^1^ Phase 1 indicates fattening phase 1 (1–3 month), phase 2 indicates fattening phase 2 (4–6 month), and phase 3 indicates fattening phase 3 (7–11 month). H = high energy and high protein diet, C = moderate energy and moderate protein diet, and L = low energy and low protein diet. ^2^ SEM, standard error of means. ^3^ P × D indicates interaction effect between fattening phase and dietary treatment.

**Table 7:** Effects of dietary nutrient density on rumen bacterial composition at genus level (relative abundance > 0.5%) in the whole fattening phases of Holstein steers.

Genus Name ^1^	Phase 1			Phase 2			Phase 3			SEM ^2^	*p* - Value ^3^		
	H	C	L	H	C	L	H	C	L		Phase	Diet	P × D
*Prevotella*	38.93	31.53	29.48	31.20	30.57	28.56	28.20	25.78	22.53	2.49	0.005	0.042	0.651
*Uncultured rumen bacterium*	14.14	14.76	14.63	14.85	14.81	15.89	15.33	16.04	15.64	1.17	0.434	0.833	0.920
*Rikenellaceae RC9 gut group*	4.63	7.96	10.83	2.80	5.34	7.85	4.35	6.29	10.06	0.83	0.001	<0.001	0.629
*Succiniclasticum*	3.60	2.96	1.74	6.68	4.25	2.75	8.74	5.98	3.94	0.48	<0.001	<0.001	0.111
*Prevotellaceae UCG-003*	3.53	3.09	4.63	1.69	3.13	4.37	1.37	2.92	3.36	0.47	0.026	0.003	0.246
*Ruminococcaceae NK4A214 group*	1.54	2.15	1.37	2.58	1.91	1.61	3.64	3.11	2.27	0.23	<0.001	0.003	0.069
*Prevotellaceae UCG-001*	3.38	2.22	1.73	4.95	1.50	1.66	2.08	1.26	1.44	0.56	0.095	0.017	0.186
*Ruminococcus 2*	2.16	1.71	0.79	2.50	1.70	0.72	1.89	2.38	0.85	0.36	0.771	0.003	0.412
*Fibrobacter*	0.86	1.12	2.38	1.04	1.51	3.01	0.38	0.45	1.26	0.20	<0.001	<0.001	0.117
*Christensenellaceae R-7_group*	0.82	1.27	1.01	0.82	1.00	1.18	1.71	1.76	1.81	0.16	<0.001	0.247	0.538
*Ruminococcaceae UCG-011*	0.56	1.28	1.17	0.60	1.10	1.51	0.41	1.55	1.90	0.19	0.109	0.001	0.099
*Lachnospiraceae NK3A20 group*	0.85	0.82	0.53	1.90	0.89	0.58	2.01	1.29	0.75	0.20	0.003	0.003	0.066
*Prevotellaceae UCG-004*	1.36	1.22	1.01	0.69	1.05	1.27	0.47	0.74	0.90	0.14	0.009	0.104	0.112
*Succinivibrionaceae UCG-002*	0.56	1.02	0.81	0.79	1.72	1.22	0.49	0.96	0.81	0.32	0.160	0.089	0.929
*Moryella*	0.99	0.87	0.38	0.91	1.30	0.55	1.18	1.24	0.85	0.15	0.054	0.003	0.484
*Ruminococcaceae UCG-014*	0.61	0.90	0.93	0.92	1.18	0.56	0.93	1.12	0.88	0.14	0.302	0.087	0.103
*Eubacterium coprostanoligenes group*	0.72	0.80	0.62	0.96	0.69	0.55	1.32	1.17	1.00	0.09	<0.001	0.025	0.440
*Veillonellaceae UCG-001*	0.44 ^b^	0.66 ^b^	0.71 ^b^	0.61 ^b^	0.67 ^b^	0.76 ^b^	1.96 ^a^	1.01 ^ab^	0.96 ^b^	0.17	0.002	0.541	0.031
*Treponema 2*	0.60	0.77	0.95	0.77	0.89	1.13	0.77	0.61	0.78	0.12	0.097	0.121	0.441
*Prevotellaceae NK3B31 group*	0.71 ^ab^	0.98 ^ab^	0.59 ^ab^	0.60 ^ab^	0.74 ^ab^	0.94 ^ab^	0.32 ^b^	0.70 ^ab^	1.27 ^a^	0.16	0.999	0.100	0.011
*Selenomonas 1*	0.77	0.68	0.61	0.88	0.98	0.52	0.91	0.70	0.51	0.11	0.352	0.045	0.247
*Candidatus_Saccharimonas*	0.37	0.56	0.50	0.32	0.74	0.63	0.83	0.97	0.80	0.08	<0.001	0.021	0.393
*Ruminococcus 1*	0.46	0.66	0.69	0.74	0.69	0.66	0.36	0.45	0.72	0.08	0.031	0.063	0.072
*CAG-352*	0.37	0.63	0.45	0.93	1.04	0.49	0.33	0.68	0.46	0.13	0.003	0.057	0.153
*Ruminococcaceae UCG-010*	0.34	0.59	0.68	0.30	0.43	0.66	0.33	0.60	0.95	0.07	0.018	<0.001	0.118
*Saccharofermentans*	0.56 ^abc^	0.68 ^ab^	0.60 ^abc^	0.37 ^cd^	0.40 ^cd^	0.41 ^bcd^	0.28 ^d^	0.56 ^abc^	0.70 ^a^	0.06	0.002	0.004	0.043

^1^ Phase 1 indicates fattening phase 1 (1–3 month), phase 2 indicates fattening phase 2 (4–6 month), and phase 3 indicates fattening phase 3 (7–11 month). H = high energy and high protein diet, C = moderate energy and moderate protein diet, and L = low energy and low protein diet. ^2^ SEM, standard error of means. ^3^ P × D indicates interaction effect between fattening phase and dietary treatment, lowercase letters are marked only when the interaction effect was significant.

## Supplementary Materials

The following are available online at https://www.mdpi.com/2076-2607/8/3/335/s1, Figure S1: Dynamic changes in ambient temperature (°C) and relative humidity (%) and temperature-humidity index semimonthly as fattening phases advanced. Figure S2: Shannon-Wiener curves based on Shannon index for high energy and high protein diet (H), moderate energy and moderate protein diet (C), and low energy and low protein diet (L).
