# Supplementary figures and images for: Digestive Ability, Physiological Characteristics, and Rumen Bacterial Community of Holstein Finishing Steers in Response to Three Nutrient Density Diets as Fattening Phases Advanced
Source: Microorganisms. 2020 Feb 27;8(3):335. doi: 10.3390/microorganisms8030335 (PMC7142484; doi:10.3390/microorganisms8030335)

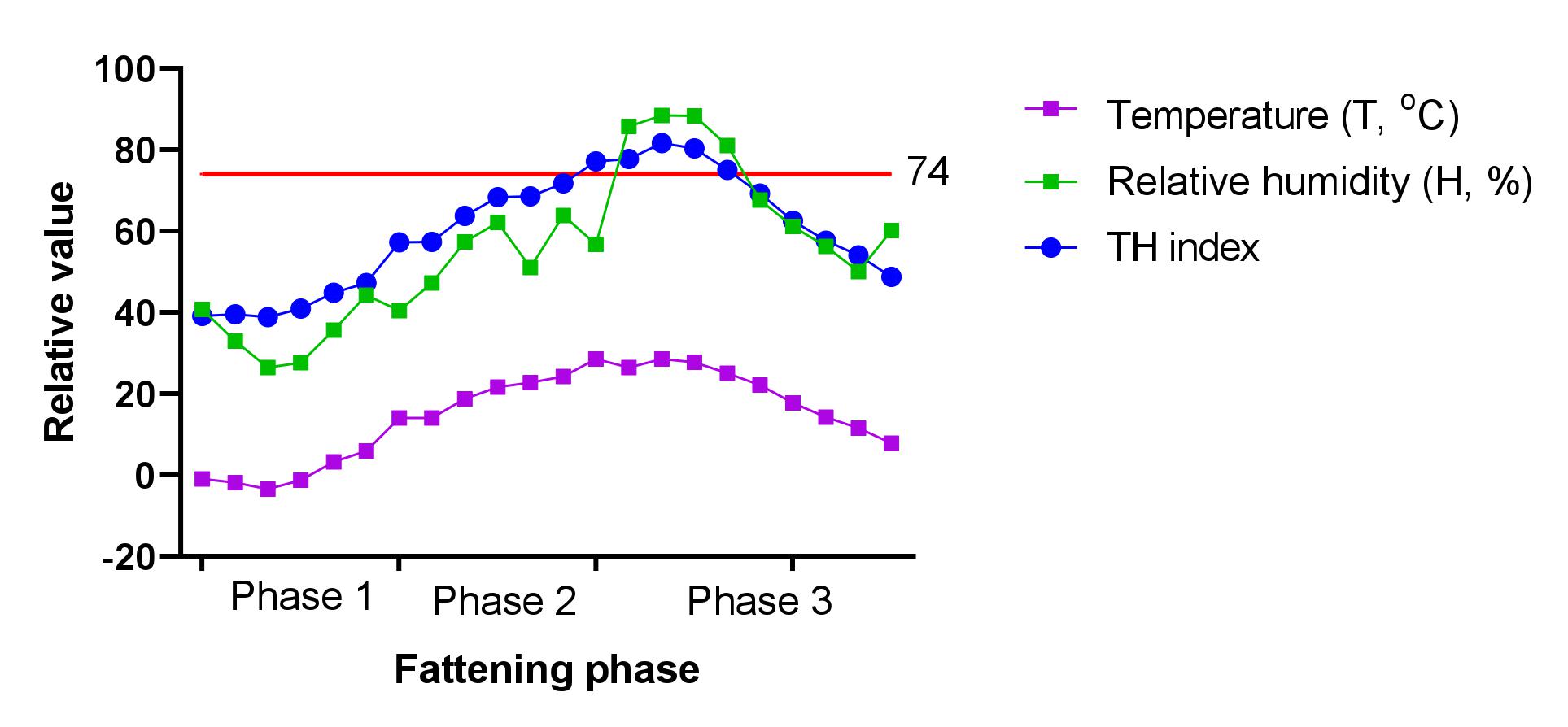

Supplement: Supplementary file 1 [file microorganisms-08-00335-s001.zip › supplementary materials/Figure S1.jpg]

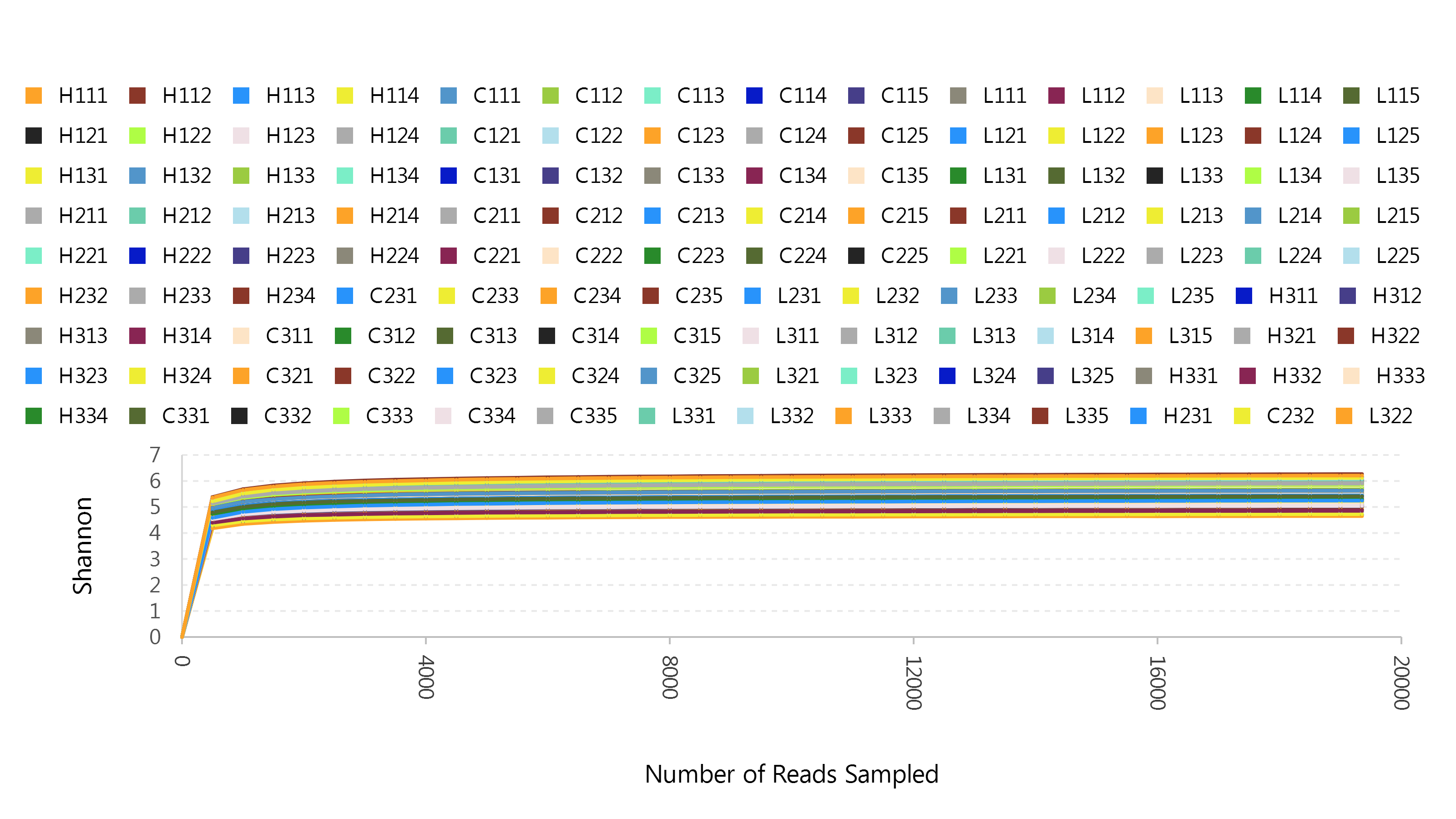

Supplement: Supplementary file 1 [file microorganisms-08-00335-s001.zip › supplementary materials/Figure S2.jpg]
